# Variation of physical wood properties and effect of dasometric variables in *Ochroma pyramidale* trees growing in plantation

**DOI:** 10.1016/j.heliyon.2024.e41210

**Published:** 2024-12-12

**Authors:** Róger Moya, Carolina Tenorio, Verónica Villalobos-Barquero, Alejandro Meza-Montoya

**Affiliations:** Escuela de Ingeniería Forestal, Instituto Tecnológico de Costa Rica, Apartado, 159-7050, Cartago, Costa Rica

**Keywords:** Balsa wood, Growth rate, Low density, Sandwich panel, Tropical species

## Abstract

Physical properties were studied in commercial plantation of balsa established in Costa Rica. Among other variables studied, physical properties varied mainly for tree age, spacing, stand density, diameter, and height of trees, which we named dasometric conditions. The aim of this study was (i) to determine the variation of specific gravity (SG), air-dry density (AD), green density (GD), and green moisture content (GMC), (ii) to know the site effect and dasometric conditions on these properties, and (iii) to establish the relationship between the four physical properties. The results showed that: SG from 0.08 to 0.21, AD from 90 to 250 kg/m^3^, GD varied 203–274 kg/m^3^, and GMC from 38.8 to 137.1 %. Tree age affected statistically all physical properties, it was positively correlated with SG, AD, and GD, but negatively correlated with GMC. Diameter breast height and total height were weakly correlated with SG and AD, respectively. Commercial height and stand density were highly correlated with SG and AD, besides stand density was positively correlated with GMC. AD was positively correlated with SG and GD but negatively correlated with GMC. According to the results, balsa tree plantations exhibited significant variation in physical properties (SG, AD, GD, and GMC) in trees aged between 30 and 40 months and this variation was primarily attributed to the site-specific growth conditions, mainly temperature and precipitation. These wood properties can be selected by site and growing conditions.

## Highlights

• Physical properties varied with age, spacing and shape of trees.
• The aim of this study was the variation of physical properties.
• Tree age was positively correlated with specify gravity and air-density.
• Diameter and total height were weakly correlated with these parameters.
• Air-density could be represented AD=23.6+718.1∗SG+0.11∗GD.

## Graphical abstract

**Figure:**
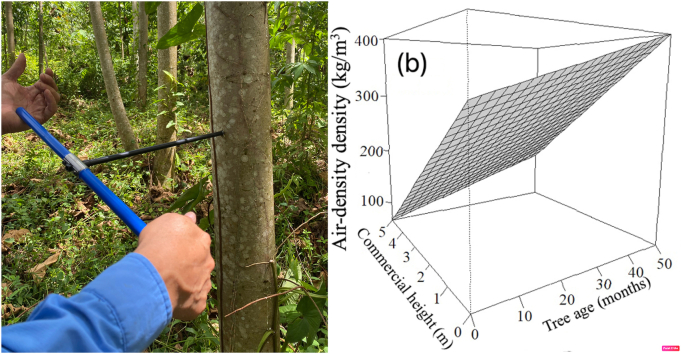

## 1 Introduction

*Ochroma pyramidale* Sw. (balsa) is a tropical timber tree species that grows naturally from southern Mexico to Bolivia, or at latitudes from 22° N to 15° S [1]. Balsa wood has a low air dried density, ranging from 50 to 350 kg/m^3^ [2]; therefore, this species has been widely used in of engineering products applied to civil infrastructure (windmills, bridges), transportation (cars, trucks, vans, trains, aircraft, boats), industrial (packing, storage) and for leisure like sports equipment and musical instruments [3].

Plantations were established under the concept of fast-growth plantations, to produce wood rapidly [1,4]. Recently, there is an increase in demand from China and wind turbine blade-producing countries [5], thus, many commercial plantations have expanded in large areas of tropical America [6]. Although there is extensive knowledge of the establishment and management of commercial balsa plantations, the experiences in management, establishment, and rotation age are rarely documented [7,8].

It is well known fact that the physical properties of wood determine its various uses [9]. Specifically, specific gravity (SG) is considered to be the most important parameter because it determines many wood properties and that can be used to predict other wood characteristics [10]. Another significant wood property related to industrial processes, particularly during drying or energy use, is green moisture content (GMC). GMC indicates the humidity level at the time of harvesting, and this water must be removed to use the wood [9]. In case of balsa wood, the most crucial physical characteristic is air-dried density (AD), which represents the density in air-dry conditions, typically 12 % [11]. This parameter is essential for manufacturing various products, such as core sandwiches [3] and end-grain panels [11].

Several factors affect the SG or AD of balsa wood [12]. SG increases from the pith to the bark in trees from natural forests, [[13], [14], [15], [16], [17]]. This variation mainly occurs in natural forests and mature trees, not in plantation trees, as natural forest conditions allow normal tree maturation [13,14]. Additionally, there is a height variation, with SG decreasing from the base to the top of the tree [15,16].

Studies on density or SG variation in plantation trees are limited, but some studies have reported the effects of tree position and growth rate [[18], [19], [20]] and different precedence [21]. Williamson and Wiemann [13,14] found no variation from the pith to the bark in the plantation. In contrast, Pertiwi et al. [19,20] reported that SG increases with growth rate and stabilizes at 8 cm in diameter in 7-year-old trees with varying growth rates (slow, medium, and fast growth). Pecegueiro et al. [21] recorded differences in SG due to different precedencies in plantations in Ecuador and that an increasing growth rate produces low SG. Listyanto et al. [22] found an SG of 0.14 in three-year-old trees, statistically lower than the SG of 0.19 in four-year-old balsa wood.

Based on the above mentioned literature, the present study is aimed to determine the variation of four physical properties (specific gravity, air-dry density, green density, and green moisture content) in four different sites in the Atlantic Zone of Costa Rica, the effects of dasometric conditions (i.e., tree age, diameter at breast height, commercial and total height, and stand density) on these physical properties, and to establish the relationship between these physical properties in *Ochroma pyramidale* trees from plantations between 28 and 42 months of age.

## 2 Materials and methods

### 2.1 Studio region

**Fig. 1:**
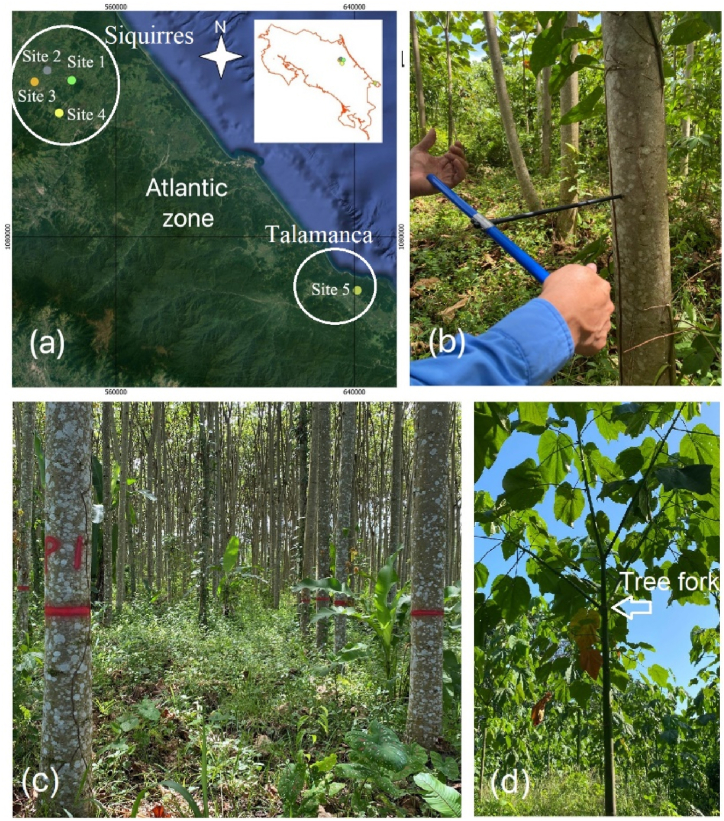
Geographic localization of five fast-growth plantations in the Atlantic zone of Costa Rica (a), increment core extraction (b), plantation in site 5 (c) and tree fork with three branches (d).

Commercial fast-growing balsa plantations are established in two regions of the Limón Province (Fig. 1a) of Costa Rica, which is approximately 100 km from one to the other. These two regions were in Siquirres and Talamanca canton and were selected because they are separated by 100 km from each other and presented different growing conditions (Fig. 1a). The Siquirres region has an average annual precipitation of 3844 mm, an average temperature ranging from 21 to 30 °C, without a defined dry period [23]. According to the classification from Holdridge's life zones [24], it presents a life zone of Tropical humid Forest. The Talamanca region presents an average precipitation of 2300 mm, an average temperature that varies from 20 to 25 °C [23] and as per Holdridge's Life Zones it belongs to very Humid Montane Forest [24].

### 2.2 Sampling from the commercial plantations

Five different commercial plantations were selected in the two regions (Siquirres and Talamanca) in the Limón Province of Costa Rica (Fig. 1a). The plantations had been established by a group of farmers (Table 1, Fig. 1c), which each site presented different site conditions, spacing, management, and weed control (Table 1). Besides these farmers, to different to others one, they helped with research and all facility to the development of research. Farmers of sites 2, 4, and 5 planted balsa trees with different spacing, then there was a different initial stand density. Farmers of sites 1 and 3 planted only with one spacing (Table 2). In general, if any thinning was applied and natural mortality occurred, then at sample time, the stand density was different (Table 1, Table 2). The weed control was applied manually in plantations, the fertilizers were applied at the moment of plantation establishment and subsequently every year at the beginning of the rainy season in May. The description of the plantation is shown in Table 1.

**Table 1:** Description of plantation and site of five fast-growth plantations of *Ochroma pyramidale*.

Site	Annual rain (mm/year)	Temperature (°C)	Total area (ha)	Nursing by	Re-planted	Fertilization	Weed control	Pruning	Thinning
1	3890	27.4	2.34	Seed	Yes	Yes	Yes	Not	Not
2	3715	27.4	5.45	Seed	Yes	Not	Yes	Not	Yes
3	3996	27.4	2.30	Seed	Yes	Not	Yes	Not	Not
4	3845	27.4	1.41	Seed	Yes	Not	Yes	Not	Yes
5	2390	26.2	168.84	Seed	Yes	Yes	Yes	Not	Not

**Table 2:** Dasometric data of five fast-growth plantations of *Ochroma pyramidale*.

Site	Plot	Age (months)	Spacing (m)	Initial Stand density (tree/ha)	Stand density at sample time (tree/ha)	Total height (m)	Diameter at breast height (cm)	Commercial height (m)
Site 1	P4	30	2.5 x2.0	2000	720	13.48	15.56	5.91
Site 2	P1	31	2.0 x3.0	1667	680	12.13	14.36	5.11
	P3	31	1.5 x2.0	3333	2320	12.94	11.40	4.21
	P4	31	1.5 x2.0	3333	1360	12.11	13.68	4.16
	P2	31	2.0 x3.0	1667	1620	12.35	14.60	5.19
	P5	31	2.0 X2.0	2500	2320	11.87	13.36	3.97
Site 3	F2	34	2.0 X2.0	2500	2080	14.13	15.66	6.5
Site 4	F1	32	2.0 X2.0	2500	1380	12.18	14.15	4.99
	P3	32	2.5 x2.5	1600	1000	12.94	11.40	4.21
Site 5	P1	40	2.0 x2.0	2500	1400	12.34	11.95	4.86
	P2	40	3.0 x3.0	1111	960	13.33	14.69	5.61
	P6	40	3.0x3.0	1111	1020	13.48	15.94	6.46
	P7	40	3.0x3.0	1111	920	14.09	17.81	6.86
	P8	40	3.0x3.0	1111	920	13.37	16.35	6.18

### 2.3 Characterization of plantations

A temporary plot with a radius of 12.62 m (500 m^2^) was established in different spacings of selected plantations. The quantity of temporary plots varied in relation to the total area of plantation (Table 2), but the quantity represented 5 % of the total area. The stand density of the plantation was determined by counting the trees present in the plot and dividing them by the area of the temporary plot (500 m^2^), using equation (1). The values were extrapolated to 1 ha (10000 m^2^). Diameter at breast height (DBH), total height, and commercial height were measured in all trees presented in the plots. DBH was measured using diameter taper and the different heights were measured using telescopic measuring poles. The apex loses its apical dominance then the tree fork is presented in balsa trees, the trunk is forked in 3 different branches (Fig. 1d), then the commercial height was measured where the tree fork was presented.

(1) $$Stand\;density\;\left(N/ha\right)=\frac{Number\;of\;tree\;in\;the\;plot}{Plot\;area=500\;\left(m2\right)\;}$$

### 2.4 Sampling of the balsa trees

Six trees near the temporal plot were randomly selected in different spacings of each site. Selected trees had a straight trunk, normal branching, and no disease or pest symptoms. A total of 170 trees were sampled and DBH for each sampled tree was measured, and commercial and total tree height was also recorded at the time of sampling. Thereafter, two increment cores of 8 mm in diameter were extracted in the direction of the pith-bark (Fig. 1b) separated by 90° in each tree, according to Williamson and Wiemann methods [14]. The increment cores were placed in plastic tubes to prevent drying and then transported to the laboratory.

### 2.5 Determination of specify gravity, green density and green moisture content

The volume (Vg) and weight (Wg) of the increment core in green condition were determined. Vg was calculated by measuring the diameter and length of the increment core. Subsequently, these cores were placed in a controlled conditions in a chamber to reach 12 % moisture content (temperature of 22 C and relative humidity of 66 %) or air-dry condition for 4 weeks. Then the samples were again weighed (W_12 %_) and their volume (V_12 %_) was determined with the dimensions of diameter and length. Followed by these samples were placed in the oven at 103 C for 24 h and then weighed again (W_0%_). Then this information was determined from the following parameters: Specify gravity (equation (2)), green density (equation (3)), green moisture content (equation (4)) and density at 12 % or air-dried density (Equation (4)).

(2) $$Specify\;gravity\;\left(SG\right)=\frac{Weight\;in\;oven-dried\;condition\;\left(W_{0\%}\right)}{Volume\;in\;green\;condition\;\left(Vg\right)\;}$$

(3) $$Green\;density\;\left(GD\right)=\frac{Weight\;in\;green\;condition\;\left(W_{g}\right)}{Volume\;in\;green\;condition\;\left(Vg\right)\;}$$

(4) $$Green\;moisture\;content\;\left(GMC\right)=\frac{Weigth\;in\;green\;condition\;\left(W_{g}\right)-Weigth\;ovendried\;condition\;\left(W_{0\%}\right)}{Weigth\;ovendried\;condition\;\left(W_{0\%}\right)}$$

(5) $$Density\;at\;12\%\;\left(AD\right)=\frac{Weigth\;in\;air-dried\;condition\;\left(W_{12\%}\right)}{Volume\;in\;air-dried\;condition\;\left(V12\%\right)\;}$$

### 2.6 Statistical analysis

● First, the normality and homoscedasticity of all measured variables were verified using the Kolmogorov-Smirnov test (K-S test).
● Second, a one-way analysis of variance (ANOVA) was applied, considering the site as the independent variable and the physical properties measured SG, AD, GD and GMC as response variables. Differences between averages of physical properties were established using the Tukey test. The variance analysis and the Tukey tests were conducted using the SAS software (SAS Institute Inc., Cary, NC).
● The effect of dasometric variables and tree age on physical properties was analyzed using the Pearson correlation matrix. Statistical significance was evaluated at a 95 % confidence level.
● Based on the results of the correlation matrix, a forward stepwise analysis was conducted to define the priority dasometric variables affecting physical properties.
● The definition of priority dasometric variables was performed as graphical support using surface analyses for different polynomial correlations to interpret the most important variable interactions.
● Finally, the relationships between SG and AD, GD, and GMC were examined, as well as the relationships between AD and GD, AD and GMC, and GD and MC. This analysis established which parameter could be used to predict AD. The correlation, stepwise analysis and surface analyses were conducted using the SAS software (SAS Institute Inc., Cary, NC).

## 3 Results

### 3.1 Physical properties of balsa

**Fig. 2:**
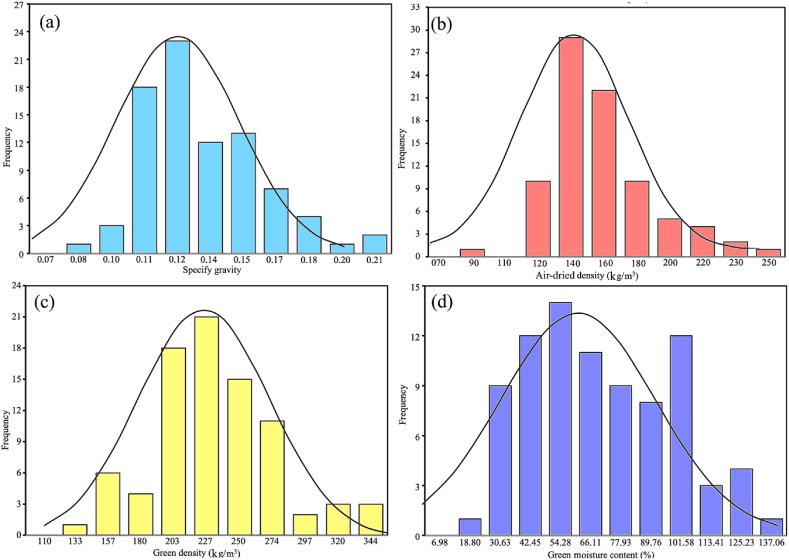
Frequency distribution for four physical properties of *Ochroma pyramidale* in trees growing in fast-growth plantations: (a) specify gravity, (b)air-dried density, (c) green density and (d) green moisture content.

SG with normal distribution (K-S test value of 0.94 and P-value<0.0001) varied from 0.08 to 0.21 and the highest was present from 0.11 to 0.15 (Fig. 2a). AD variation ranged from 90 to 250 kg/m^3^ with a normal distribution (K-S test value of 0.93 and P-value<of 0.0001) and the most important frequency was presented between 120 kg/m^3^ to 180 kg/m^3^ (Fig. 2b). GD presented a variation from 133 to 344 kg/m^3^ and the range from 203 kg/m^3^ to 274 kg/m^3^ was the highest frequency (Fig. 2c) and the data presented normality distribution (K-S test value of 0.95 and P-value of 0.04). The variation of GMC was from 18.80 % to 137.06 %, the highest frequency was from 30 to 102 % (Fig. 2d), and the values of normality were K-S test value of 0.94 and P-value<0.0001, this physical parameter presented a normal distribution.

### 3.2 Site effect on physical properties

Table 3 presents the site effect on the physical properties evaluated in the present investigation. Statistically, In SG and AD parameters, the highest statistical values were recorded in site 4 and site 5, and no differences between the two were observed. Other three sites, the AD was the lowest (Table 3). Site 1 presented the lowest GD values, whereas other four sites (from site 2 to site 5) no statistical difference was observed. The GMC was the highest in site 1 and site 3, followed by sites 2 and 4, and the lowest value was observed in site 5.

**Table 3:** Physical properties variation by site for four physical properties of *Ochroma pyramidale* in trees growing in fast-growth plantation.

Site	Specify gravity	Air-dried density (kg/m^3^)	Green density (kg/m^3^)	Green moisture content (%)
Site 1	0.12^A^ (10.35)	128^A^ (10.83)	156^A^ (11.53)	98.67^A^ (9.94)
Site 2	0.12^A^ (14.78)	135^A^ (10.27)	205^B^ (16.40)	85.15^B^ (38.31)
Site 3	0.12^A^ (11.73)	139^A^ (10.74)	217^B^ (10.11)	106.67^A^ (12.46)
Site 4	0.14^AB^ (15.97)	150^AB^ (11.74)	238^B^ (17.80)	73.06^B^ (33.73)
Site 5	0.16^B^ (19.19)	167^B^ (22.61)	232^B^ (20.32)	48.86^C^ (27.74)

Legend: Different letters for each physical property means statistical difference at 99 % level of confidence. The values between parentheses represent the coefficient of variation.

### 3.3 Effect of dasometric condition on physical properties

Statistically, tree age affected all physical properties, and it was positively correlated with GD, SG, and AD, but negatively correlated with GMC (Table 4). The DBH and total height were positive and weakly correlated with AD and SG, respectively. Commercial height and stand density were highly correlated with SG and AD, besides stand density was also positively corrected with GMC (Table 4).

**Table 4:** The person Correlation coefficient between physical properties and dasometric parameters of *Ochroma pyramidale* in trees growing in fast-growth plantation.

Physical properties	Tree age	Diameter breast height	Commercial height	Total height	Actual stand density
Specific gravity	0.555∗∗	0.211^NS^	0.339∗∗	0.226∗	−0.538∗∗
Air-dried density	0.470∗∗	0.246∗	0.353∗∗	0.140^NS^	−0.380∗∗
Green density	0.291∗∗	0.014^NS^	0.093^NS^	0.094^NS^	−0.207^NS^
Green moisture content	−0.664∗∗	−0.111^NS^	−0.158^NS^	−0.078^NS^	0.520∗∗

Legend: ∗∗ statistically significant at 99 % of confidence level, ∗ statistically significant at 99 % of confidence level and NS not significant.

Forward stepwise regression showed that tree age was the variable that has the highest influence on the four physical properties evaluated. Interestingly, commercial height was the second variable that contributed to AD and GMC variation. Similarly, DHB appointed a few variations of GMC parameters. Although stand density affected statistically GMC and SG, the contributions were weak, with less than 6 % of the variation of these physical properties (Table 5).

**Table 5:** Regression forward stepwise analysis for physical properties and dasometric parameters of *Ochroma pyramidale* in trees growing in fast-growth plantation.

Physical properties	Variable	Step	Estimated parameter	Proportion R^2^ multiple	R^2^ multiple
Specify gravity	Intercept	0	0.115∗	–	–
	Tree age	1	0.00259∗∗	0.3079	0.3079
	Stand density	2	−0.00339∗∗	0.0572	0.3651
Air-dried density	Intercept	0	309.46∗∗	–	–
	Tree age	1	1.823∗∗	0.2202	0. 2202
	Commercial height	2	29.01∗	0.0441	0.2643
Green density	Intercept	0	136.66∗∗	–	–
	Tree age	1	3.66∗∗	0.0880	0.0880
	DBH	2	−3.23 ^NS^	0.0153	0.1033
Green moisture content	Intercept	0	178.56∗∗	–	–
	Tree age	1	−4.98∗∗	0.4408	0.4408
	Commercial height	2	21.94∗∗	0.0882	0.5289
	Stand density	3	0.015∗∗	0.0444	0.5734

Legend: ∗∗ statistically significant at 99 % of confidence level, ∗ statistically significant at 99 % of confidence level and NS not significant.

**Fig. 3:**
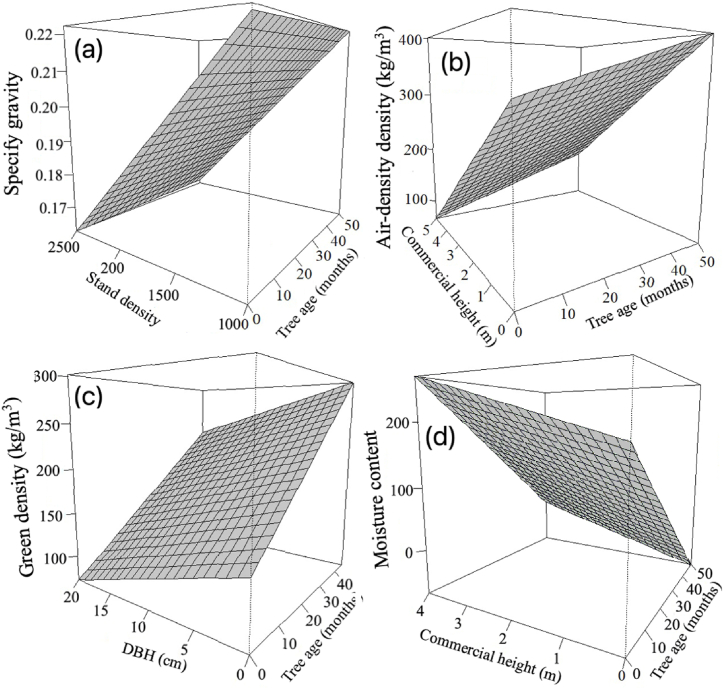
Surface plane variation of (a) specify gravity (b) air density, (c) green density, and (d) green moisture content in relation to tree age and dasometric variables.

Effect of tree age and dasometric variables' on four physical properties are presented in the surface plane (Fig. 3). The surface plane of SG increased with age and decreased with stand density, so the plane was highest at higher age and low densities, but the lowest point occurred at lower ages and with the highest stand densities (Fig. 3a). Although AD and GD increased with tree age, DBH showed an opposite effect, i.e., there is a decrease in these parameters with the increase in DBH and commercial height, respectively. Thus, the surface plane is higher at increase in ages of the trees but lowers DBH or commercial height. In contrast there is a decline in these parameters with increasing DBH or commercial height and at lower age (Fig. 3b and c) of trees. GMC has a different surface plane; it reached its maximum when the commercial heights is higher and tree ages is low. However, it begins to decrease with the increasing age of the trees at low commercial heights (Fig. 3d), due to tree age affected GMC negatively whereas commercial height affected positively.

### 3.4 Relationship between air-dried density and other physical properties

**Fig. 4:**
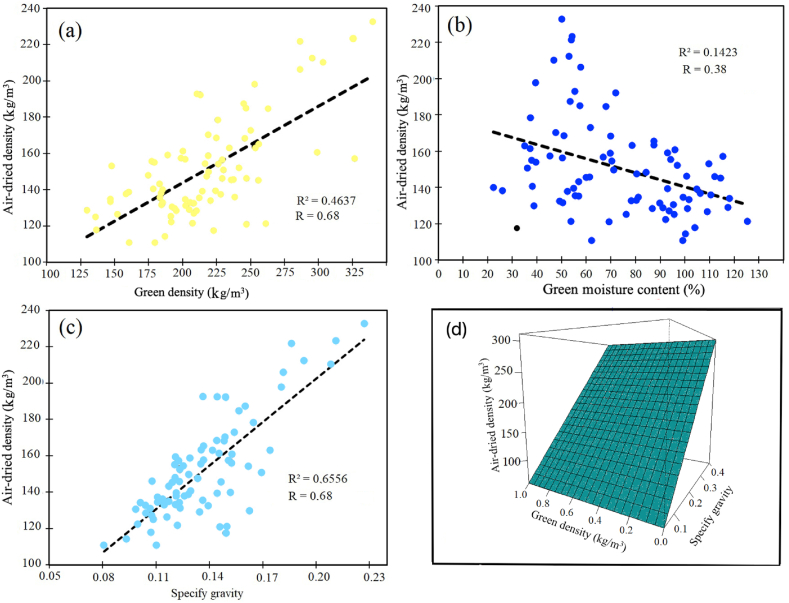
Relationship between physical properties of *Ochroma pyramidale*: (a) green density vs air-dried density, (b) green moisture content vs green density and (c) specify gravity vs air-dried density.

AD was positively correlated with SG and GD (Fig. 4a and c) but negatively correlated with GMC (Fig. 4b). Forward stepwise regression showed that SG was the variable with the highest influence on AD with 65 % of the total variations, followed by GD with 2.35 % of the variation, and moisture content did not present the effects on AD variation (Table 6). Then AD could be modelled with the following relationship and its relationship is represented in Fig. 4d.

(5a) Air−drieddensity=23.56+718.12∗SG+0.109∗Greendensity

**Table 6:** Regression forward stepwise analysis for air-dried density and specify gravity, green density and green moisture content of *Ochroma pyramidale* in trees growing in fast-growth plantation.

Variable	Step	Estimated parameter	Proportion R^2^ multiple	R^2^ multiple
Intercept	0	23.56 ^NS^	–	–
SG	1	718.12∗∗	0.6547	0. 6547
Green density	2	0.109∗	0.0235	0.6782
MC	3	0.091 ^NS^	0.0042	0.6824

Legend: ∗∗ statistically significant at 99 % of confidence level, ∗ statistically significant at 99 % of confidence level and NS not significant.

## 4 Discussion

The variation in the physical properties of the wood was substantial (Fig. 2). The most significant amplitude occurred in GMC, which included seven high-frequency categories without a well-defined mode (Fig. 2d). In contrast, the other physical properties SG, AD and GD presented the greatest amplitude in only four categories with a well-defined mode (Fig. 2a–c). Some important aspects to note are: (i) the variation in these properties is typical and species specific [25,26], and the variation in the physical properties observed in this study is consistent with this trend (Fig. 2). (ii) This variation in physical properties may affect industrial processes for the products of end users [27,28]. For instance, high variation in GMC negatively impacts the drying process, which results in less uniformity in the target moisture content [29]. For balsa wood, this variation could affect the drying time, and may lead to irregular time for drying and less uniformity in the moisture content of different boards. As the total weight for transport is calculated based on GD, it may also affect transport of wood logs [30,31]. Therefore, these variations in GD can hinder accurate total weight calculations.

As mentioned earlier, AD is the most crucial physical parameter in balsa wood [11] due to its significance in fabricating many products [3,11]. Four categories are considered for different products: light (80–120 kg/m3), medium (120–180 kg/m^3^), heavy density (180–220 kg/m^3^), and AD values over 220 kg/m^3^, which are not considered optimal for commercial purposes. The ideal density for the commercialization of balsa wood is between 100 and 170 kg/m^3^ [32,33]. The high frequency (i.e., 120–220 kg/m^3^) of AD in the present study, and the lower frequency values over 220 kg/m^3^ suggest that wood from the fast-growth plantations can be commercialized internationally, with only a small quantity being unsuitable for the international market.

Comparing the results obtained in the present study with those of other studies is complex. Most reported values are derived from natural forest trees, and there is often confusion between basic density and SG [14]. However, many studies report values of AD, which is the condition under which the wood is marketed [31]. Despite these challenges, comparisons will be attempted. The SG values in the present study ranged from 0.08 to 0.21 (Fig. 2b), that are consistent with the values reported by Listyanto et al. [22], who found SG values of 0.14 for 3-year-old trees and 0.19 for 4-year-old trees from fast-growth plantations. Similarly, the range of values found in this study aligns with those reported by earlier researchers (Williamson and Wiemann [13,14], Wiemann and Williamson [17], Baker [18], Rueda and Williamson [16], and Whitmore [15]) for 5-7-year-old plantations in Costa Rica, which showed a variation of 0.10–0.20.

AD values varied from 120 to 220 kg/m^3^ (Fig. 2c), which agreed with the values reported by Jenkin et al. [7] in Papua New Guinea, Pertiwi et al. [19,20] in 7-year-old plantations in East Java, Uzcátegui-Rojas et al. [32] in Venezuela, Silva et al. [33], Christensen-Dalsgaard and Ennos [12] in 12-month-old plantations, Avram et al. [34], and Pecegueiro et al. [21] in Brazil. A limited literature is available on other physical properties (such as GD and GMC) evaluated in this study. For example, Avram et al. [34] reported GD values of 148–250 kg/m^3^ for balsa plantations, consistent with the present study's findings, which showed a variation of 133–320 kg/m^3^ (Fig. 2a). These authors also mentioned that the GMC for the species was 85 %, like the present study's values, which ranged from 31 to 125 % (Fig. 2d).

The ecological conditions of the different sites also showed its effects on wood formation in different species [35]; thus, balsa is not an exception (Table 3). It is observed that there was little variation between sites 1 to 4 in the evaluated properties, but site 5 presented important differences in the properties as compared to sites 1–4 (Table 3). This difference may be due to sites 1–4 being in the Siquirres region and site 5 being in the Talamanca region, which presents different climatic conditions. As compared to site 5 which is located in Talamanca, the first site presents a higher average temperature and precipitation than. Site 5 is characterized bya drier climate and lower temperature, which in general, presented the higher values in the parameters related to the mass, which are GD, AD, and SG; therefore, it represents the lower GMC. Dry sites generally produce high SG as a means of survival in low water conditions, unlike areas with higher humidity that present more favourable conditions for survival. Unfavorable conditions produced higher SG values, which is a key trait for plant growth, survival, and forest carbon storage [35]. This behaviour agreed with studies of Christensen-Dalsgaard and Ennos [12] and Whitmore [15]. Christensen-Dalsgaard and Ennos [12] found a lower density (from 97.3 to 101.6 kg/m^3^) in well-watered sites than in droughted sites (117.1–122.2 kg/m^3^) in 12-month-old trees, values too in concordance with this study. Whitmore [15] found that high variation in average density due to environmental conditions, as expressed in terms of the three lowland regions of Costa Rica. The highest density was found in dry sites, especially Pacific dry, and the lowest density was presented in the Atlantic Region of Costa Rica.

Dasometric conditions and tree age affected physical properties (Table 4); however, tree age was the parameter that had the strongest influence on these properties’ (Table 5). Present study showed that tree age has positive effect on SG, AD, and GD (Fig. 3a–b, d), but showed a negative impact on GMC (Fig. 3c). The increase in density with increase in age can be attributed to that of anatomical elements, specifically a decrease in ray frequency due to the concentration of auxins [36]. Availability of the growth hormones is the main components that regulate cambium cell divisions, which in turn decreases with increasing age. As observed in fast-growing balsa trees from plantations; this results in low vessel and ray frequencies [37], and causes an increase in density with tree age, (Fig. 3a–b, d).

The second dasometric parameter with the most significant effects on physical properties was commercial height that directly affected the AD and GMC (Table 6, Fig. 3b and c). Although commercial height is not crucial for the wood properties of many commercially planted species, it did have an impact on physical properties of balsa wood's (Table 4, Fig. 3b and c). This effect can be attributed to the presence of stem forks in balsa trees. The stem form of balsa trees is associated with the formation of stem forks, notably those resulting from multileader whorls at around 12 months of age [9]. After a tree forks, its diameter begins to increase [38], affecting properties associated with tree growth, including SG and anatomical features [39,40]. Therefore, since commercial height was measured at the point where the tree fork occurred, physical properties were negatively affected.

Other dasometric parameters, specifically DBH and stand density, negatively impacted GD and SG. An increase in these parameters (Table 4, Fig. 3a–d) reduced GD and SG. Stand density affects competition between trees, resulting in reduced growth at high stand densities and better diameter development and growth rates at low stand densities [41]. Thus, at high stand density, trees produce smaller stem diameters, whereas low stand density increases stem diameter and growth rate. Typically, a higher growth rate decreases SG and GD values [41]. This behaviour differs from what was observed in balsa trees, where low stand density produced a high growth rate and increased SG (Fig. 3d). This finding is similar to the study by Pertiwi et al. [19,20], which found that an increase in growth rate increases SG.

According to Glass and Zelinka [9], the prediction of AD can also be determined with SG and oven-dry density. Without this determination, the oven-dry volume must be measured, which often requires ensuring the wood does not absorb water, making the process complex due to the need for paraffin coating, which complicates measurement. AD can also be calculated using more comprehensive mathematical equations [9]. Thus, the relationship found in the present study provides a quick way to determine AD using parameters that can be measured quickly and with greater precision.

## 5 Conclusion

Balsa plantations exhibited significant variation in physical properties (SG, AD, GD, and GMC) in trees aged between 30 and 40 months in the Atlantic region of Costa Rica. This variation was primarily ascribed to the site-specific growth conditions, mainly temperature and precipitation. Trees from the drier sites produced wood with higher SG, AD, and GD, but lower GMC as compared to trees from the wetter sites. Secondly, the age of the plantation had the highest influence on the physical properties. In contrast, management conditions of the plantation had a lesser impact, particularly on commercial height, stand density, and DBH. The occurrence of stem forks at an early age has direct influence on the commercial height, due to formation of forks at low heights. Stand density affected the SG, with an unusual trend where low stand density (from 1000 to 200 trees per hectare) produced the highest SG values. Therefore, careful management is necessary to avoid producing wood with excessively high density, which can limit the species' commercial viability.

In general, trees from commercial plantations before 41 months of age produced wood with appropriate density (less than 220 kg/m^3^) for commercial purposes. For commercial wood, it is necessary to know the air-dried density, which can take several days for its measurement. According to this study, it is possible to determine this value using other physical properties that are determined in 24 h, such as SG and GD. This air-dry density relationship can be modelled as follows: AD=23.6+718.1∗SG+0.11∗GD.

## CRediT authorship contribution statement

**Róger Moya:** Writing – review & editing, Writing – original draft, Validation, Methodology, Investigation, Data curation, Conceptualization. **Carolina Tenorio:** Writing – original draft, Methodology, Investigation, Formal analysis, Conceptualization. **Verónica Villalobos-Barquero:** Methodology, Data curation, Conceptualization. **Alejandro Meza-Montoya:** Data curation, Conceptualization.

## Data availability

All data presented in this article were deposited in: https://dataverse.tec.ac.cr/dataset.xhtml?persistentId=doi:10.18845/RDA/AWJIUM.

## Funding sources

This work was supported by the Vicerrectoría de Investigación y Extensión del Instituto Tecnológico de Costa Rica.

## Declaration of competing interest

The authors declare that they have no known competing financial interests or personal relationships that could have appeared to influence the work reported in this paper.
